# Surgically Managed Acquired Vulvar Lymphangioma Circumscriptum

**DOI:** 10.4314/ejhs.v32i1.25

**Published:** 2022-01

**Authors:** Getu Dinku Heyi, Sintayehu Debas Endalew, Tadie Siraw Mulu, Mulu Melkie Gesese

**Affiliations:** 1 College of Medicine and Health Sciences, Tibebe Ghion Specialized Hospital, Bahir Dar university; 2 Felege Hiwot specialized Hospital

**Keywords:** Lymphangioma circumscriptum, vulva, vulvectomy

## Abstract

Lymphangioma circumscriptum is a rare benign disorder of lymphatic channels in deep dermal and subcutaneous layers. It can occur either as a congenital abnormality or as acquired damage to previously normal lymphatic channels. It occurs in different parts of the body, and the vulva is one of the parts which is commonly affected. Here, we presented a 39 years old para 2 who presented with vulvar swelling. She was diagnosed with acquired lymphangioma circumscriptum of the vulva and superficial vulvectomy was done.

## Introduction

Lymphangioma circumscriptum is a benign disorder of lymphatic origin that can be either congenital or acquired, involving the skin and subcutaneous tissues. Acquired vulvar lymphangioma circumscriptum is a rare disease known to occur after radical hysterectomy with or without adjuvant radiation therapy for cervical cancer, chronic lymphedema of the lower extremity, or infections like Tuberculosis. It is caused by damage to previously normal lymphatic channels (1,2,3).

The clinical presentation of lymphangioma circumscriptum is highly variable, ranging from being asymptomatic to a highly disabling condition. Discomfort, itching, rubbing, and lymph oozing are the most frequent symptoms. Clinically, it usually presents as multiple nodular lesions, verruciform or polypoid, with variable size and with intervening normal skin (1,5).

It may pose diagnostic difficulties both clinically and histopathologically. It is frequently confused with warts or condyloma acuminate. The main differential diagnosis is genital warts (2,4,5). There are different modalities of management, of which laser, ablative therapy, and surgery, and for asymptomatic cases, observation are options of management with variable risk of recurrence (1,2,3).

Here, we present a 39 years old para 2 woman who presented with vulval swelling. She was diagnosed with acquired lymphangioma circumscriptum and for whom superficial vulvectomy was done. There was no recurrence over 9 months of follow-up.

## Case Report

A 39 years old para 2, both vaginal deliveries, who had regular menstrual cycle presented with vulval swelling of two years duration with swelling. The patient reported a progressive increment of the swelling, oozing and itching sensations. She also reported swelling of her right leg of 2 years duration. Five years back, she underwent laparotomy for an unspecified gynecologic disorder which looked benign condition.

On physical examination, she had an old infra umbilical midline scar, but there was no other finding on abdominal examination. On genital examination, bilaterally swollen labia with multiple popular and vesicular lesions with some oozing of watery discharge from the mass, the vulvar skin over the mass was shiny. Other genital examinations were none reviling. On musculoskeletal examination, she had non-pitting edema of the right extremity upto the thigh.

For her condition, after repetitive visits to different OPDs, she was diagnosed with lymphangioma circumscriptum at a dermatology clinic, and she was linked to the gynecology department for management. Before her final diagnosis, she was diagnosed with vulval eczema, genital wart. Vulvar cancer was considered and treated for eczema and genital wart, but no improvement and biopsy were made before her final diagnosis.

Her basic investigations were nonrevealing; pelvic ultrasonography was normal. After evaluation at the gynecology department, she was counseled on options of management and potential recurrence of the lesion. Even after surgical management, she was admitted for elective surgery after written consent and bilateral superficial vulvectomy was done with primary skin closure done under spinal anesthesia.

After surgery, she was followed at gynecology and dermatology OPDs. She had small oozing from the wound site at 9 O clock until 27th postoperative day with some swelling of the clitoris, but the itching and pain had subsided and on her 40th day of postoperative follow-up day, the wound had healed well with no recurrence of the lesion and no discharge. She was linked to the internal medicine side for follow-up of lower extremity lymphedema. At the 9th month follow-up, she did not have recurrence on the vulva, and she was wearing elastic bandages on the lower extremity. She also reported that she was having sexual intercourse without difficulty.

Pathologic examination described scattered arteriolar size dermal and subcutis vessel, exocrine and apocrine ducts involved (cuffed) by intense mononuclear infiltrate noted. A few dilated lymphatic lumens filled with lymphatic fluid were noted. For the above description, the pathologist concluded vulva; nonspecific chronic inflammation with minimal dermal lymphedema accumulation.

## Discussion

Vulvar lymphangioma circumscriptum sometimes accompanies lower extremity edema or genital lymphedema. It may pose diagnostic difficulties both clinically and histopathologically. It is frequently confounded with warts or condyloma acuminate. The most common dermoscopic pattern associated with lymphangioma circumscriptum is the presence of lacunae and vascular structures (1,5).

This patient had a risk factor of lower limb lymphedema, but her diagnosis was challenging. She had visited different OPDs to reach a final diagnosis and she had taken different medications for her condition, but the final diagnosis was reached at the dermatology clinic. Her pathology report description of few dilated lymphatic lumens filled with lymphatic fluid which favors diagnosis of vulvar lymphangioma circumscriptum.

Acquired lymphangioma occurs more commonly in the vulvar region compared to the other regions of the body, which can be frequently associated with surgery, radiation therapy, infection (erysipelas, tuberculosis, etc.), Crohn's disease, congenital dysplastic angiopathy, and congenital lymphedema (1,2,4,5). It is a frequently suggested hypothesis for the etiology of lymphangioma circumscriptum that superficial lymphatics of the skin cannot develop lymphatic connections in the deep layers. They are small clear herpetiform pseudovesicles. Vesicles may be localized with clear borders or diffuse across a larger area or form groups. Primary lymphangioma stems from local malformations of lymphatics and manifests itself early in life. On the other hand, acquired lymphangioma develops secondary to chronic obstruction of lymphatics and can manifest itself at any age. (2,4)

Even though this patient did not have the commonest risk factor of pelvic radical surgery or radiation to her pelvis or treatment history for tuberculosis, she presented with a typical clinical picture. For this case, the only risk factor was the presence of lower limb lymphedema. Her previous pelvic surgery was for benign pathology, which evidences do not mention as it increases the risk of having lymph angioma circumscriptum of vulva.

There is no standard management for vulvar lymphangioma circumscriptum. The various treat-ments range from conservative treatment with decon-gestive physiotherapies, such as manual lymph drainage, exercise, and compression, to abrasive therapy, sclero-therapy, electrocoagulation, laser-therapy with CO2, and surgical excision depending on the patients' condi-tions. While lesion recurrence is frequent, out of various treatment modalities, surgical excisions were preferred. The recurrence rate after surgical management was 23.1% on a follow-up ranging from 6–81 months. The recurrence rate might be twice as high in lymphangioma circumscrip-tum without surgical management. The recurrence rate after radical excision was high when the initial lesions were greater than 7 cm diameter as compared with lesions of less than 7 cm diameter for which a local excision was performed (1,2,4,5).

In our case management, we choose superficial vulvectomy for the reason of lack of other management modalities and the extensive nature of the lesion. She was symptomatic which made her not an ideal candidate for conservative management. For nine months of follow-up, she had no recurrence of the lesion. Due to the rarity of vulvar lymphangioma circumscriptum, it is common to misdiagnose the condition timely which will force patients to get treatment for the misdiagnosed condition, which will make delay in giving timely and appropriate management of the condition. Therefore, high index of suspicion is mandatory for better management.

## Figures and Tables

**Figure 1:**
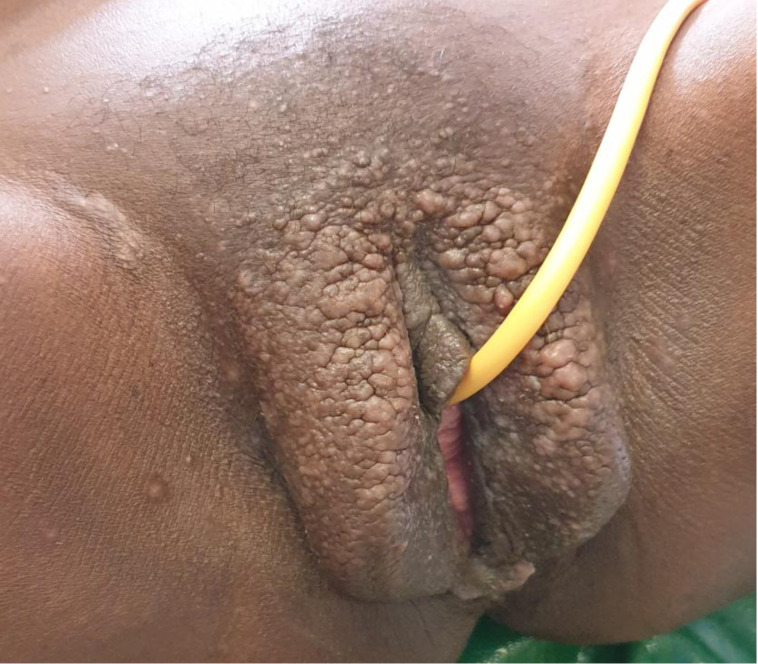
picture of vulvar lymphangioma circumscriptum before surgery

**Figure 2:**
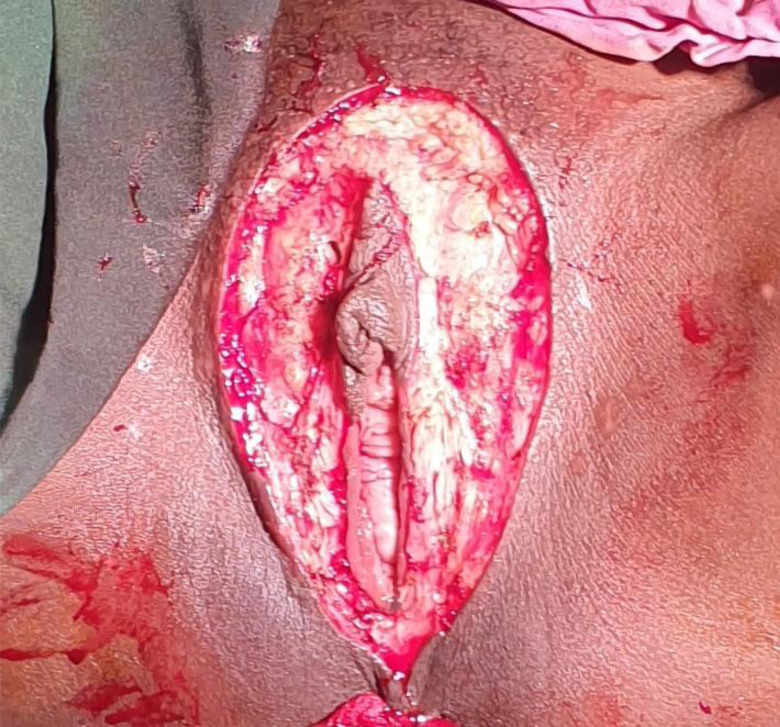
After vulvectomy before skin approximation

**Figure 3:**
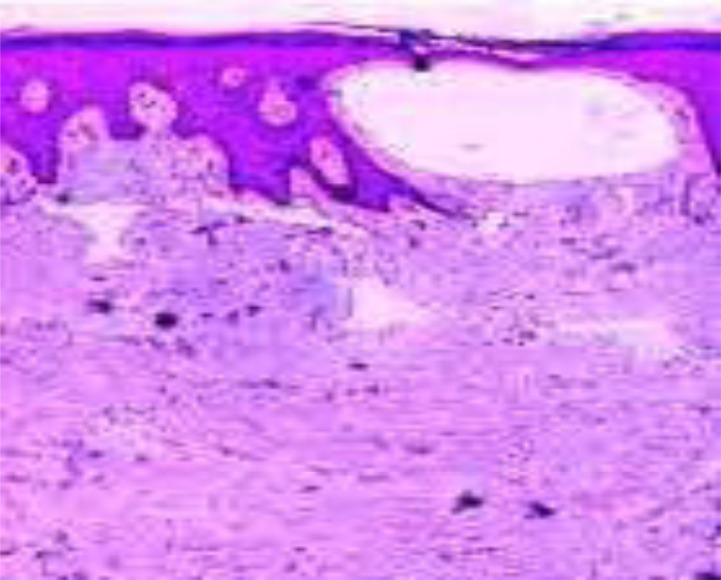
Histologic picture
